# The Observing Physician

**Published:** 1888-04

**Authors:** 


### *THE OBSERVING PHYSICIAN.*

Dr. Bedford Brown, of Alexandria, in delivering the President's address before the Medical Society of Virginia, at its late session, said: No physician can become a successful student of the constitution and diseases of his race who does not cultivate assiduously habits of observation. It must be a law of his life. When we cease to be observers, we inevitably fall into habits of routinism. Probably no class of men see more of the mysteriously shifting phases of nature, or have a wider field for observation and a fitter one to profit by those means, than the practicing physicians. In truth, every physician, to be successful, must depend largely on what he observes at the bedside. This is the grand theatre for medical observation; for the books, with all their profound and comprehensive learning, guide us only over a portion of the way, and then leave a vast field, dark, unexplored, and full of mystery, in our travels, through which we must depend largely upon our own good sense and personal observations. And it is in this unexplored region of medical science that the practical student most needs his powers of observation. There exists stored up in the mind of every practicing physician, acquired by personal observation in his silent watching of disease, an unwritten experience that must be of incalculable value to himself, upon which he is at liberty to call on all occasions. Every good practitioner possesses a stock of this unwritten experience, which is always ready and convenient for application to practical use. Every man is a learner and can be a teacher on occasions. Every intelligent practitioner could, if so disposed, give to the profession useful and valuable information, derived from his own observation and experience, which no other possesses. But it is sadly true that some men never observe. Some observe superficially, while others are profound and analytical observers of everything that comes within their mental range. It is the latter who accumulate valuable and useful information from every source, and who become conspicuous as men of resource and practical worth.

